# Patient-psychiatrist discordance and drivers of prescribing long-acting injectable antipsychotics for schizophrenia management in the real-world: a point-in-time survey

**DOI:** 10.1186/s12888-022-03846-x

**Published:** 2022-03-17

**Authors:** Alexander Keenan, Dee Lin, Jason Shepherd, Hollie Bailey, Carmela Benson, Sophie Meakin

**Affiliations:** 1 Janssen Global Services, LLC, Titusville, NJ USA; 2 Janssen Scientific Affairs, LLC, Titusville, NJ USA; 3 Adelphi Real World, Bollington, UK

**Keywords:** Schizophrenia, Long-acting injectable, Antipsychotics, Patient-physician communication, Prescribing drivers, Discordance

## Abstract

**Background:**

To assess discordance between psychiatrists and their patients with schizophrenia regarding disease management and understand drivers of prescribing long-acting injectable (LAI) antipsychotics.

**Methods:**

Data were collected via the Adelphi Schizophrenia Disease Specific Programme™, a point-in-time real-world international survey of psychiatrists and their consulting patients with schizophrenia, conducted in 2019. Psychiatrists completed an attitudinal survey on schizophrenia management and provided patient profiles for their next 10 adult consulting patients. The same patients voluntarily completed patient self-completion forms. Disease severity and improvement were assessed via physician-reported Clinical Global Impression scale; patients’ adherence to treatment was rated through a 3-point scale (1=not at all adherent, 3=fully adherent).

**Results:**

Four hundred sixty-six psychiatrists provided data for 4345 patients (1132 receiving a LAI; 3105 on non-LAI treatment; 108 not on treatment). LAIs were more commonly prescribed to patients with severe schizophrenia, with varying reasons for prescribing. Globally, only slight agreement was observed between psychiatrists and patients for Clinical Global Impression severity of illness (κ=0.174) and level of improvement on treatment (κ=0.204). There was moderate agreement regarding level of adherence to treatment (κ=0.524). Reasons for non-adherence did not reach a level of agreement greater than fair.

**Conclusions:**

Our real-world survey found that LAIs were more often reserved for severe schizophrenia patients and improving adherence was a key driver for prescribing. However, compared with the patients themselves, psychiatrists tended to underestimate patients’ disease severity and overestimate their adherence.

**Supplementary Information:**

The online version contains supplementary material available at 10.1186/s12888-022-03846-x.

## Background

Schizophrenia is a chronic psychiatric disorder estimated to affect more than 21 million people worldwide; it is associated with severe disease burden and poor psychosocial outcomes. While many treatments have become available for its management, the disorder still presents substantial challenges to providers, caregivers, and the healthcare system [1].

Effective treatment of mental health conditions, such as schizophrenia, requires a patient-centred approach. Shared decision-making has been recommended in schizophrenia management to optimize treatment regimens and improve adherence. Indeed, patient-physician trust and communication are important to help drive positive outcomes [2], and patient-physician alliance and communication have been found to be influential on patients’ level of adherence to treatment [3, 4], with the odds of patients’ adherence being 1.6 times higher with good communication [5]. One study found that shared understanding is particularly important in schizophrenia: increased patient participation in checking understanding of what the psychiatrist is saying and correcting misunderstandings were associated with better adherence to treatment after six months [3]. In schizophrenia, there may be a breakdown in the patient-psychiatrist communication and the discordance around treatment goals between psychiatrists and patients tends to be due to psychiatrists focusing on more traditional treatment goals, such as reduced psychotic symptoms, improved self-confidence and reduced mistrust/hostility, whereas patients tend to value more tangible concepts, such as improved satisfaction, activities of daily living and capacity for work; such discordance between psychiatrists and their patients presents a barrier to communication and providing patient-centred care. Therefore, concordance of treatment goals is essential for shared decision-making [6].

As one key challenge in schizophrenia is non-adherence to medications, especially in patients taking daily oral antipsychotics, which was reported in around 50% of patients [7], early engagement in discussions about appropriate therapy is particularly important. Consequences of non-adherence include relapse, hospitalisation, higher risk of suicide, poorer prognosis, drug and alcohol consumption, poor mental performance, and low satisfaction with life [8].

While many patients with schizophrenia struggle with adherence to antipsychotics that require daily oral intake, patients who are prescribed long-acting injectable (LAI) antipsychotics have considerably better adherence [8], with reported rates as high as 97.7% in patients receiving intramuscular antipsychotics [9]. Moreover, compared with oral antipsychotics, the use of LAIs is associated with many other advantages, such as lower relapse rates, less probability for rebound symptoms and rapidly occurring/abrupt relapses, and a reduction in the rate of hospitalisation [10, 11]. A recent study in a real-world setting found that LAI treatment reduced the readmission rate by 29% compared with oral medication; furthermore, LAIs reduced the readmission rate by 58% in patients with repeated admissions [11]. Another real-world study found that second-generation LAIs reduced hospitalisation rates and emergency room (ER) visits, improving the economic burden of schizophrenia [12]. Lastly, a systematic review and meta-analysis of real-world studies, which investigated rates of hospitalisation and ER visits, as well as treatment adherence among patients with schizophrenia in the United States (US), found that compared with patients initiated on an oral agent, patients initiated on LAIs were less likely to have an ER visit, had fewer all-cause ER visits and were more likely to be adherent to their medication [13].

Furthermore, a recent systematic literature review of 19 international schizophrenia clinical practice guidelines found that all except one discussed the use of LAIs for patients with schizophrenia and 74% of them recommended LAIs for patients who are non-adherent to other antipsychotic administration routes. The latest guidelines from the American Psychiatric Association [14] recommends LAIs when patients are transitioning between inpatient and outpatient settings, while other guidelines [15, 16] position second-generation LAIs as an initial treatment option after sufficient efficacy and tolerability has been established with oral formulation of the same antipsychotic agent [17]. However, despite the benefits of LAI antipsychotics and the guideline recommendations, these agents are underutilised in clinical practice due to a combination of patients’ and psychiatrists’ beliefs and attitudes, as well as service barriers, which can affect best practice and evidence-based care in LAI prescribing [18].

The aims of our survey were to: 1) assess discordance between psychiatrists and their patients with schizophrenia regarding disease management; 2) understand the drivers of prescribing LAIs to patients with schizophrenia in a real-world clinical setting.

## Methods

### Study design

Data were collected via the Adelphi Schizophrenia Disease Specific Programme (DSP™), a point-in-time survey of psychiatrists and their consulting patients with schizophrenia in real-world clinical practice. DSP methodology has been previously described and validated [19–21]. Data were collected in the US, France, Spain, Japan, and China between July and December 2019.

### Participants

Psychiatrists were identified via local fieldwork partner physician panels and publicly available lists and were invited to participate in the DSP following the completion of a short screening questionnaire. Eligible psychiatrists were actively involved in treatment decisions for patients with schizophrenia and saw at least five adult patients with schizophrenia in a typical week. Participating patients were aged ≥18 years at data collection, had a psychiatrist-confirmed diagnosis of schizophrenia, and were not participating in a clinical trial at the time.

### Data collection

Participating psychiatrists were divided into two cohorts based on their LAI prescribing status, defined by responding “yes” (LAI prescribers) or “no” (LAI non-prescribers). All psychiatrists completed a survey on their attitudes towards and perceptions of schizophrenia treatment and management. The survey was translated into local languages, as appropriate. Examples of survey questions are provided in Supplementary Table S1 (Additional file 1).

Psychiatrists then completed electronic patient record forms (PRFs) for their next 10 consulting adult patients with schizophrenia, of whom eight were outpatients and two were inpatients (where possible). The sample size for each psychiatrist (*n*=10) was chosen following an assessment of what would be feasible to collect and the 8:2 split was to ensure that an adequate number of inpatients was captured for analysis.

The same patients were invited to voluntarily complete a patient self-completion form (PSC). Both PRFs and PSCs included questions about patients’ clinical characteristics, severity of illness, treatment patterns and outcomes.

### Outcomes

Disease severity and improvement were based on physician-reported Clinical Global Impression (CGI) scale on current treatment [22]. The CGI is a brief three-item physician-rated scale that assesses severity of illness, global improvement or change, and therapeutic response. Physicians assessed patients’ adherence to treatment with a subjective 3-point scale, where 1 was not at all adherent (<50% of prescribed dose) and 3 was fully adherent.

### Ethics

Data collection was undertaken in line with the European Pharmaceutical Marketing Research Association guidelines [23] and as such it did not require ethics committee approval. The questionnaires used in the Schizophrenia DSP were reviewed and given exemption by the Western Institutional Review Board (reference number: AG8618). The survey was performed in full accordance with relevant legislation at the time of data collection, including the US Health Insurance Portability and Accountability Act 1996 (United States Department of Health & Human Services Summary of the HIPPA Privacy Rule 1996) and the Health Information Technology for Economic and Clinical Health Act legislation [24].

Data were collected according to market research guidelines; hence, no source validation was possible or required. Using a checkbox, patients provided informed consent to take part in the survey. Data were collected in such a way that patients and physicians could not be identified directly. Physician and patient data were pseudo-anonymized. A code was assigned when data were collected. Upon receipt by ARW, data were pseudo-anonymized again to mitigate against tracing them back to the individual. Data were aggregated before being shared with the subscriber and/or for publication.

### Analysis

Means and standard deviations were calculated for continuous variables, and frequency counts and percentages for categorical variables. For the first objective (i.e., assessing discordance between psychiatrists and their patients with schizophrenia regarding disease management), Kappa analysis was used to calculate the level of agreement between psychiatrists and patients (Supplementary Table S2, Additional file 2). A weighted Kappa was used to take into consideration the different levels of agreement between categories.

For the second objective (i.e., understanding the drivers of prescribing LAIs to patients with schizophrenia in a real-world clinical setting), patients were grouped for analysis as “LAI” and “non-LAI”; this was defined as patients receiving a LAI as part of their treatment at data collection vs. those not receiving a LAI at that time. For these bivariate analyses, the type of test employed depended on the distribution of the variable: T-tests were used for numeric variables, Mann-Whitney U tests were used for numeric variables where T-test assumptions were violated, Fisher’s exact tests were used for binary categorical variables and Chi-squared tests were used for categorical variables with more than two groups.

Data were analysed for the countries combined (i.e., aggregated global data) and by individual country using descriptive statistics. Results were derived from matched PRF-PSC pairs. Any patient with missing data for a particular variable was removed from all analyses involving that variable. However, patients who were removed from one set of analysis were still eligible for inclusion in other analyses. Analyses were performed using Stata 15.1 [25].

This publication presents aggregated global data and results were interpreted at a global level. In addition, reference to individual countries is made, as appropriate, to highlight country-specific differences. Both global and individual country data are shown in the tables and figures.

## Results

A total of 466 psychiatrists participated in the survey. Most of these (*n*=389, 83.5%) were LAI prescribers; 77 (16.5%) were non-prescribers. Overall (global level), LAI prescribers spent significantly less time seeing patients in a hospital setting than non-prescribers (mean 51.1% vs. 75.6% of their time, *p*<0.0001). Significantly more LAI prescribers than non-prescribers had also been involved in clinical trials in the past (45.5% vs. 28.6%, *p*=0.0157).

### Patient demographics and characteristics

Psychiatrists provided data for 4345 patients with schizophrenia (US: 1204, France: 802, Spain: 811, China: 996, Japan: 532). Of these, 4237 (97.5%) patients were on treatment at data collection (US: 1135, France: 786, Spain: 802, China: 996, Japan: 518). Overall, 1132 (26.7%) patients were receiving a LAI and 3105 (73.3%) patients were on non-LAI treatment. Global and individual country patient demographics and characteristics in each treatment group are presented in Table 1.

**Table 1:** Patient demographics and characteristics

	Overall		US		France		Spain		Japan		China	
	LAI	Non-LAI	LAI	Non-LAI	LAI	Non-LAI	LAI	Non-LAI	LAI	Non-LAI	LAI	Non-LAI
Number of patients	**1132**	**3105**	**251**	**884**	**335**	**451**	**410**	**392**	**84**	**434**	**52**	**944**
Age, years, mean (SD)	38.9 (13.3)	39.4 (14.4)	38.8 (14.3)	40.4 (16.2)	38.5 (13.3)*	40.5 (14.3)	38.4 (12.4)	38.7 (13.8)	45.6 (14.0)	45.3 (14.4)	34.7 (10.5)	35.5 (11.7)
Gender: male, n (%)	733 (64.8)***	1706 (54.9)	149 (59.4)	516 (58.4)	222 (66.3)	276 (61.2)	285 (69.5)	250 (63.8)	50 (59.5)*	205 (47.2)	27 (51.9)	459 (48.6)
BMI, kg/m^2^, mean (SD)	26.4 (4.8)***	25.4 (4.7) (*n*=3103)	27.8 (5.4)	27.3 (4.8)	25.7 (4.5)	25.7 (4.8) (*n*=449)	26.7 (4.4)	26.2 (5.2)	25.1 (4.6)***	23.2 (4.1)	24.8 (3.4)	24.1 (3.5)
Unable to finish education due to schizophrenia, n (%)	352 (39.6)*** (*n*=888)	733 (28.4) (*n*=2579)	99 (51.0)** (*n*=194)	254 (38.1) (*n*=666)	149 (54.2) (*n*=275)	186 (52.3) (*n*=356)	86 (29.3)* (*n*=294)	113 (38.3) (*n*=295)	10 (13.7) (*n*=73)	42 (11.3) (*n*=373)	8 (15.4)	138 (15.5) (*n*=889)
Living circumstance***		(*n*=3104)										(*n*=943)
Lives with relative(s), n (%)	520 (45.9)	1481 (47.7)	103 (41.0)	363 (41.1)	111 (33.1)	150 (33.3)	247 (60.2)	243 (62.0)	39 (46.4)	254 (58.5)	20 (38.5)	471 (50.0)
Lives with partner/spouse, n (%)	197 (17.4)	827 (26.6)	41 (16.3)	201 (22.7)	44 (13.1)	68 (15.1)	63 (15.4)	65 (16.6)	21 (25.0)	67 (15.4)	28 (53.9)	426 (45.2)
Lives with friend(s), n (%)	30 (2.7)	63 (2.0)	13 (5.2)	140 (15.8)	7 (2.1)	5 (1.1)	6 (1.5)	6 (1.5)	2 (2.4)	3 (0.7)	2 (3.9)	1 (0.1)
Lives alone, n (%)	237 (20.9)	474 (15.3)	42 (16.7)	140 (15.8)	127 (37.9)	187 (41.5)	50 (12.2)	46 (11.7)	18 (21.4)	84 (19.4)	0 (0.0)	17 (1.8)
Lives in group or supported housing, n (%)	97 (8.6)	160 (5.2)	36 (14.3)	91 (10.3)	29 (8.7)	23 (5.1)	28 (6.8)	20 (5.1)	4 (4.8)	19 (4.4)	0 (0.0)	7 (0.7)
Lives in homeless shelter/Homeless, n (%)	25 (2.2)	39 (1.3)	12 (4.8)	30 (3.4)	6 (1.8)	6 (1.3)	7 (1.7)	3 (0.8)	0 (0.0)	0 (0.0)	0 (0.0)	0 (0.0)
Other, n (%)	26 (2.3)	60 (1.9)	4 (1.6)	11 (1.2)	11 (3.3)	12 (2.7)	9 (2.2)	9 (2.3)	0 (0.0)	7 (1.6)	21 (2.2)	2 (3.9)
**Change in living circumstance in previous 12 months, n (%)**	78 (7.9)** (*n*=984)	141 (5.0) (*n*=2806)	23 (12.2) (*n*=189)	57 (7.9) (*n*=721)	30 (10.2) (*n*=295)	35 (8.6) (*n*=407)	21 (5.7) (*n*=370)	13 (3.5) (*n*=368)	3 (3.8) (*n*=78)	11 (2.8) (*n*=398)	1 (1.9)	25 (2.7) (*n*=912)
**Employment status**	**	(n=3101)	**									(*n*=940)
Employed, n (%)	244 (21.6)	815 (26.3)	62 (24.7)	300 (33.9)	62 (18.5)	96 (21.3)	78 (19.0)	85 (21.7)	28 (33.3)	140 (32.3)	14 (26.9)	194 (20.6)
Unemployed, n (%)	743 (65.6)	1833 (59.1)	162 (64.5)	467 (52.8)	241 (71.9)	305 (67.6)	265 (64.6)	245 (62.5)	47 (56.0)	224 (51.6)	28 (53.9)	592 (63.0)
Other, n (%)	145 (12.8)	453 (14.6)	27 (10.8)	117 (13.2)	32 (9.6)	50 (11.1)	67 (16.3)	62 (15.8)	9 (10.7)	70 (16.1)	10 (19.2)	154 (16.4)
**Has a caregiver, n (%)**	559 (50.9)* (*n*=1098)	1654 (54.9) (*n*=3011)	88 (37.3)** (*n*=236)	221 (26.1) (*n*=848)	173 (52.0)* (*n*=333)	191 (43.4) (*n*=440)	226 (56.5) (*n*=400)	235 (60.4) (*n*=389)	24 (30.8) (*n*=78)	95 (24.2) (*n*=393)	48 (94.1) (*n*=51)	912 (96.9) (*n*=941)
**Has a professional caregiver, n (%)**	118 (21.1)*** (*n*=559)	195 (11.8) (*n*=1654)	27 (30.7) (*n*=88)	62 (28.1) (*n*=221)	39 (22.5) (*n*=173)	45 (23.6) (*n*=191)	46 (20.4)** (*n*=226)	26 (11.1) (*n*=235)	5 (20.8) (*n*=24)	19 (20.0) (*n*=95)	1 (2.1) (*n*=48)	43 (4.7) (*n*=912)
**Hours per week receiving care, mean (SD)**	30.3 (34.8)*** (*n*=551)	39.1 (36.5) (*n*=1647)	28.8 (26.8) (*n*=84)	36.2 (40.6) (*n*=218)	15.6 (15.7) (*n*=170)	19.8 (30.1) (*n*=188)	38.5 (42.8) (*n*=225)	35.5 (38.8) (*n*=235)	19.3 (16.4) (*n*=24)	25.8 (23.5) (*n*=94)	52.9 (38.2) (*n*=48)	46.1 (35.1) (*n*=912)
**Time since diagnosis, years, mean (SD)**	10.8 (10.5)*** (*n*=743)	6.3 (9.8) (*n*=2243)	10.3 (12.0)* (*n*=107)	7.8 (10.5) (*n*=445)	11.4 (10.2) (*n*=239)	11.8 (10.9) (*n*=294)	11.5 (9.8) (*n*=303)	11.3 (11.0) (*n*=297)	15.8 (13.0)* (*n*=42)	11.8 (12.1) (*n*=270)	0.9 (0.8)	0.7 (0.9) (*n*=937)
**Number of hospitalisations**												
In the past 12 months, mean (SD)	0.7 (1.3)*** (*n*=1072)	0.4 (0.9) (*n*=2968)	0.9 (1.7)*** (*n*=229)	0.5 (1.2) (*n*=805)	0.8 (1.7)** (*n*=316)	0.6 (1.0) (*n*=419)	0.5 (0.8)* (*n*=402)	0.4 (0.7) (*n*=384)	0.5 (0.7)	0.4 (0.8)	0.5 (0.6)	0.4 (0.6)
Since diagnosis, mean (SD)	3.0 (3.4)*** (*n*=486)	1.6 (2.9) (*n*=1496)	2.5 (3.7)* (*n*=67)	1.5 (3.3) (*n*=211)	3.7 (4.0) (*n*=133)	3.8 (5.4) (*n*=172)	3.1 (3.1) (*n*=223)	2.6 (3.1) (*n*=209)	2.0 (2.6)	1.2 (1.8)	1.6 (1.6)***	0.9 (1.2)

*p≤0.05; **p≤0.01; ***p≤0.0001

Blank cells indicate no change in sample size from the overall number of patients presented at the outset of the table. Where sample size varies, ‘n’ number is stated

*BMI* body mass index, *LAI* long-acting injectable, *SD* standard deviation, *US* United States

#### Demographics

Overall, compared with non-LAI patients, LAI patients were significantly more likely to be male and had a higher body mass index (BMI) (*p*<0.0001, in both cases).

#### Clinical characteristics

Overall, LAI patients had a significantly longer period since diagnosis on average vs. non-LAI patients (*p*<0.0001). This significant difference between LAI and non-LAI patients was also observed in the US (10 years LAI vs. 8 years non-LAI patients, *p*=0.031). LAI patients had experienced significantly more hospitalisations in the previous 12 months vs. non-LAI patients at global level (0.7 vs. 0.4, *p*<0.0001), in the US (0.9 vs. 0.5, <0.0001), France (0.8 vs. 0.6, *p*=0.005) and Spain (0.5 vs. 0.4, *p*=0.03). LAI patients had also experienced significantly more hospitalisations since diagnosis at global level (3.0 vs. 1.6, *p*<0.0001), in the US (2.5 vs. 1.5, *p*=0.034), and in China (1.6 vs. 0.9, *p*<0.0001).

#### Psychosocial characteristics

##### Education

Overall, there were significantly more LAI patients than non-LAI patients who were unable to finish education due to schizophrenia (*p*<0.0001). This was also significantly more common in LAI than non-LAI patients in the US (51.0% vs. 38.1%, *p*=0.002), although the opposite was observed in Spain (29.3% LAI patients vs. 38.3% non-LAI patients, *p*=0.024).

##### Housing

Overall, there was a significant difference in housing circumstances between LAI and non-LAI patients, with more LAI patients living alone, or in group or supported housing, and fewer living with a spouse or partner than non-LAI patients (*p*<0.0001); the same was also observed in China (*p*<0.0001). Furthermore, significantly more LAI patients vs. non-LAI patients had a change in their housing circumstances in the previous 12 months (*p*=0.0029).

##### Employment

At global level, significantly more LAI patients than non-LAI patients were unemployed (*p*=0.0005); the same was also observed in the US (64.5% LAI vs. 52.8% non-LAI, *p*=0.004).

##### Caregiver support

Overall, it was significantly less common for LAI patients than non-LAI patients to require a caregiver (*p*=0.024) and more common for this requirement to be for fewer hours per week on average (*p*<0.0001). However, where LAI patients had a caregiver, it was significantly less common for this to be a partner/spouse (*p*<0.0001) and significantly more common for this to be a professional caregiver vs. non-LAI patients (*p*<0.0001). Significantly more LAI vs. non-LAI patients had a caregiver in France (52% LAI vs. 43.4% non-LAI, *p*=0.02) and the US (37.3% LAI vs. 26.1% non-LAI, *p*=0.001). In Spain, where patients did have a caregiver, it was significantly more common for this to be a professional caregiver for LAI vs. non-LAI patients (20.4% LAI vs. 11.1% non-LAI, *p*=0.007).

### Level of concordance between psychiatrists and patients

#### Severity of illness

**Fig. 1:**
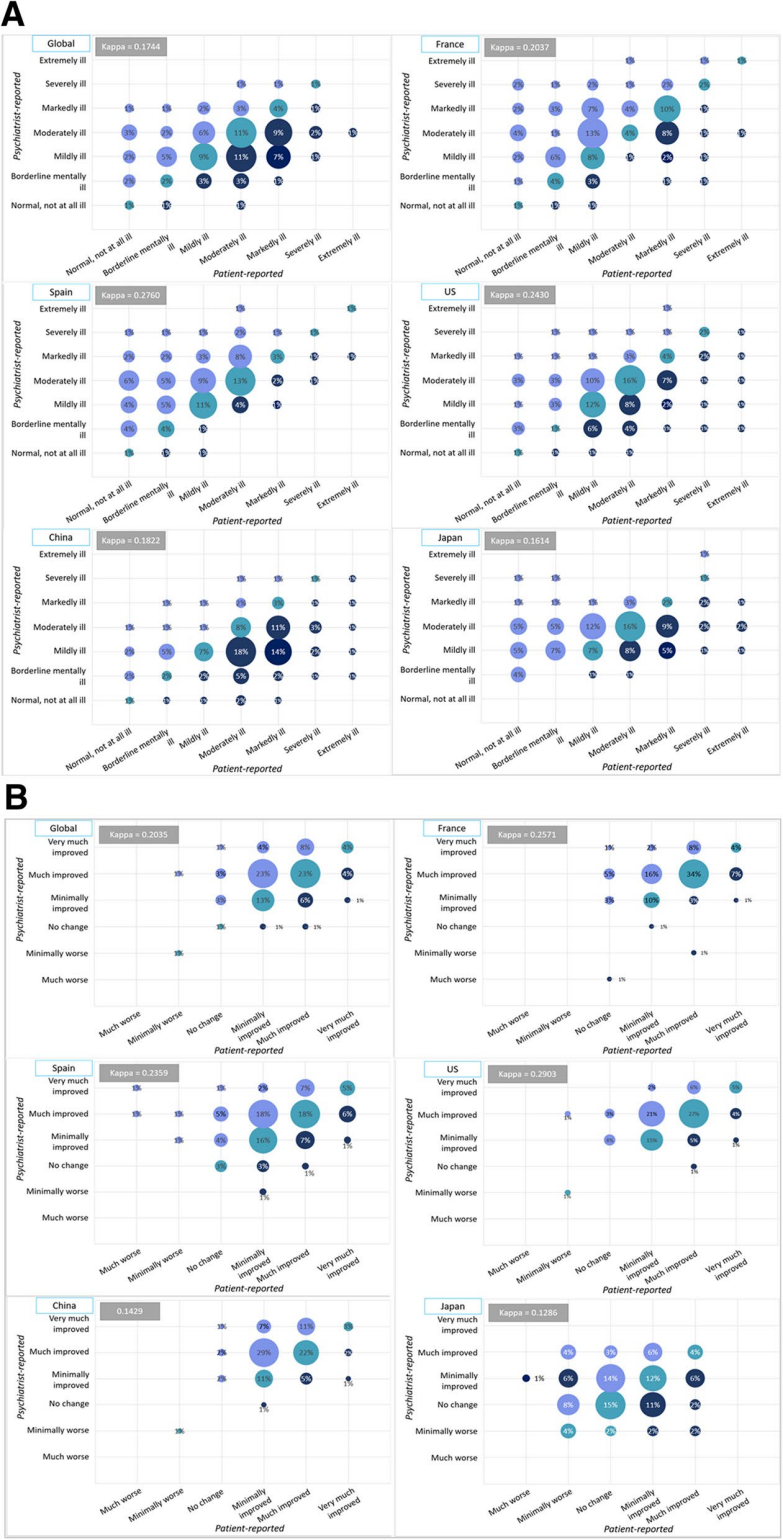
**a** Psychiatrist- vs. patients-reported CGI severity of illness. CGI: Clinical Global Impression; US, United States **b**. Psychiatrist- vs. patients-reported CGI level of improvement, CGI: Clinical Global Impression; US, United States

Globally, only slight agreement was observed between psychiatrists and their patients for patients’ CGI severity of illness (κ=0.174, Fig. 1a). Psychiatrists generally underestimated patients’ severity of illness, reporting patients as “markedly”, “severely” or “extremely ill” less frequently than patients themselves (16.6% of patients were reported as “markedly–extremely ill” according to psychiatrists vs. 30.3% patient-reported).

Only slight agreement between psychiatrists and patients was observed in Japan (κ=0.161) and China (κ=0.182) for severity of illness, while fair agreement was noted in the US (k=0.243), France (k=0.204), and Spain (k=0.276). Psychiatrists underestimated disease severity in the US, China, and Japan; the opposite was seen in France (37.6% of patients were reported as “markedly”, “severely” or “extremely ill” according to psychiatrists vs. 28.9% patient-reported) and Spain (26.9% psychiatrist-reported vs. 10.2% patient-reported).

#### Level of improvement on treatment

Only slight agreement was observed overall between psychiatrists and their patients for patients’ CGI level of improvement on treatment (κ=0.204, Fig. 1b). Psychiatrists generally overestimated patients’ level of improvement on treatment, reporting patients as “much–very much improved” more frequently than patients themselves (72% of patients were reported as “much–very much improved” according to psychiatrists vs. 46.9% patient-reported).

Only slight agreement was observed in China (κ=0.143) and Japan (κ=0.129), while fair agreement was noted in the US (k=0.290), France (k=0.257), and Spain (k=0.236). Physicians overrating improvement of illness was a consistent theme across all countries.

#### Level of adherence

**Fig. 2:**
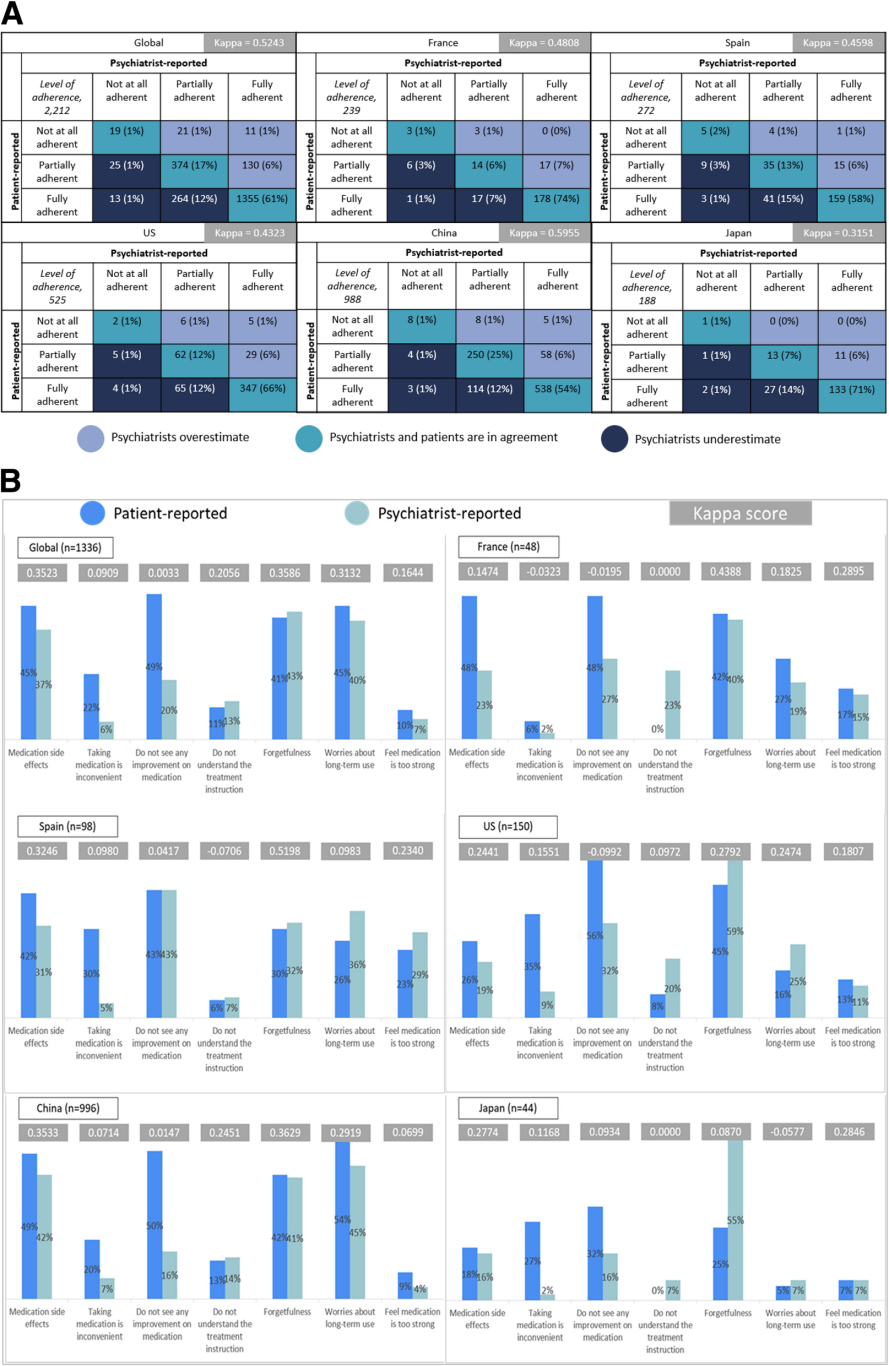
**a** Psychiatrist- vs. patient-reported adherence to schizophrenia treatment US, United States. **b** Psychiatrist- vs. patient-reported reasons for non-adherence to schizophrenia treatment US, United States

Globally, psychiatrists and patients showed moderate agreement regarding patients’ level of adherence to treatment (κ=0.524, Fig. 2a). Psychiatrists reported fewer patients as being fully adherent than patients themselves (67.6% psychiatrist-reported vs. 73.8% patient-reported). Moderate agreement was observed across all countries except for Japan, where there was only a fair agreement (κ=0.315). The highest agreement was observed in China (k=0.596).

#### Reasons for non-adherence

None of the reasons for non-adherence reached a level of agreement between psychiatrists and patients that was greater than fair (i.e., Kappa score 0.21-0.40, Fig. 2b).

Overall, where patients were not always adherent, psychiatrists tended to under-report “taking medication is inconvenient” and “do not see improvement on medication” as key reasons for non-adherence compared with patients. Psychiatrists and patients had only slight agreement on “taking medication is inconvenient” as a reason for patients’ non-adherence (κ=0.091). Psychiatrists reported this as a reason for patients’ non-adherence less frequently than patients (6.4% psychiatrist-reported vs. 22.4% patient-reported). Consistently low levels of agreement were observed across all countries, with the lowest agreement in France (κ=-0.032) and the highest in the US (κ=-0.155).

Similarly, psychiatrists and patients showed only slight agreement on “do not see any improvement on medication” as a reason for non-adherence (κ=0.003). Psychiatrists reported this as a reason for non-adherence less frequently than patients (20.1% psychiatrist-reported vs. 49.4% patient-reported). Consistently low levels of agreement were observed across all countries, with the lowest agreement in the US (κ=-0.0992) and the highest in Japan (κ=0.0934). The only exception to this pattern was in Spain, where psychiatrists and patients reported “do not see any improvement on medication” as a reason for non-adherence equally as often (42.9% in both groups).

### Drivers of prescribing

#### Reasons for prescribing current treatment and switching previous treatment

**Fig. 3:**
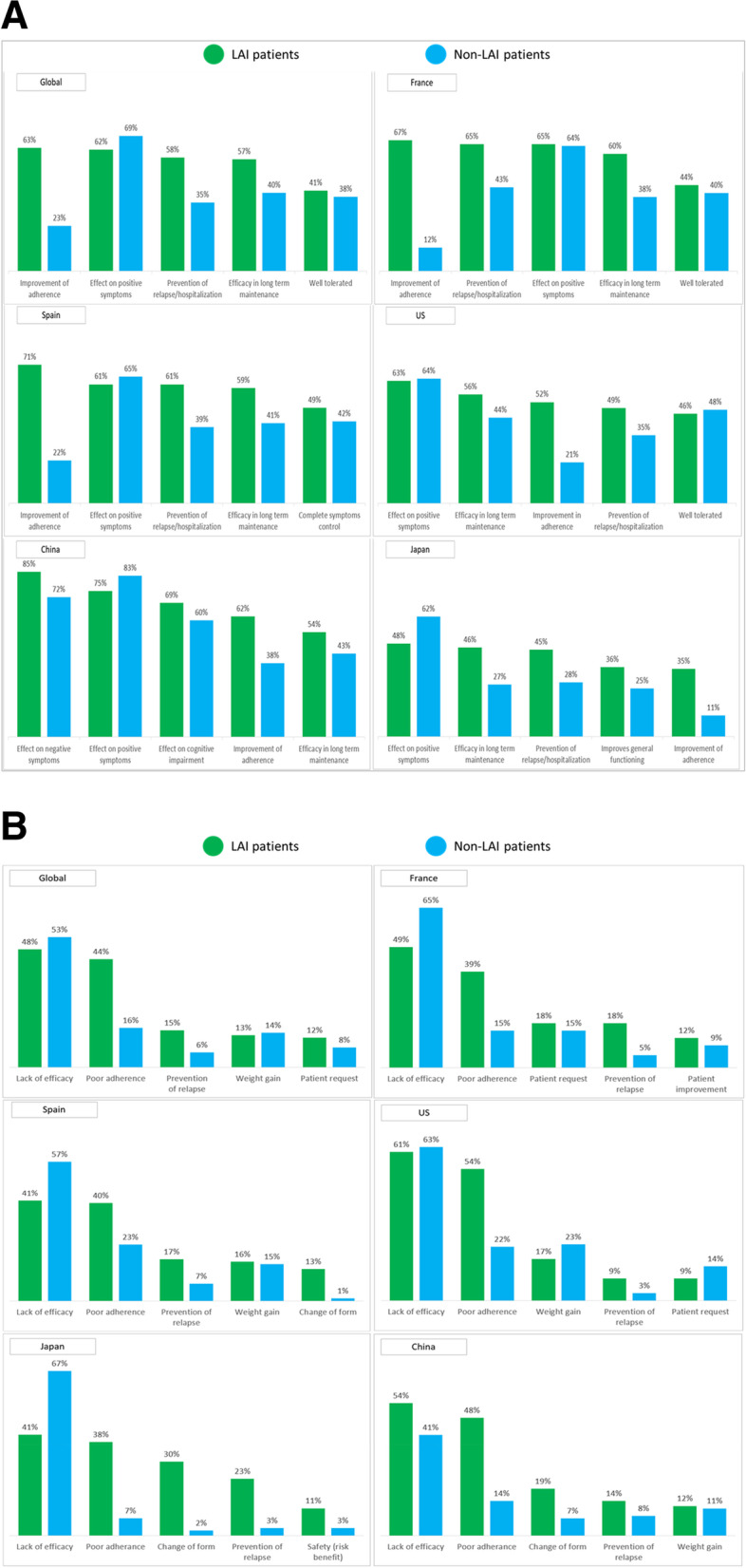
**a** Most common psychiatrist-reported reasons for prescribing patients’ current schizophrenia treatment. LAI: long-acting injectable; US, United States **b** Most common psychiatrist-reported reasons for switching patients’ previous schizophrenia treatment LAI: long-acting injectable; US, United States

Globally, the most common psychiatrist-reported reasons for prescribing LAIs over non-LAIs included improvement of adherence, prevention of relapse/hospitalisation, and efficacy in long-term maintenance (Fig. 3a). Specifically, improvement of adherence to treatment was the most frequently recorded reason for choosing LAIs and was recorded by psychiatrists significantly more often for LAIs than non-LAIs (62.7% LAI vs. 23.2% non-LAI, *p*<0.0001). Prevention of relapse/hospitalisation was also in the top 3 most frequently recorded reasons for choosing LAIs; this was recorded as a reason for significantly more LAIs than non-LAIs (57.6% LAI vs. 35.3% non-LAI, *p*<0.0001). Similarly, efficacy in long-term maintenance was recorded as a reason for more than half of LAIs, which was significantly more frequent than for non-LAIs (57.1% LAIs vs. 39.9% non-LAI, *p*<0.0001). Effect on positive symptoms (e.g., hallucinations, delusions) was also a frequently reported reason for choosing LAIs, although this was chosen by psychiatrists significantly more frequently for non-LAIs than for LAIs (62.3% LAI vs. 68.9% non-LAI, *p*<0.0001).

Improvement of adherence was a key reason for prescribing LAIs in the US, France, and Spain. Prevention of relapse/hospitalisation was a key reason in France, Spain, and Japan, and efficacy in long-term maintenance in the US and Japan.

Overall, psychiatrists reported poor adherence (43.6% LAI vs. 16.5% non-LAI, *p*<0.0001) and prevention of relapse (15.5% LAI vs. 6.0% non-LAI, *p*<0.0001) as key reasons for switching to a LAI from previous treatment (Fig. 3b**)**; this was consistent across all countries. The highest proportion of LAI patients who were switched from previous treatment due to poor adherence was in the US (54.0%) and the lowest in Japan (37.5%). The highest proportion of LAI patients who were switched from previous treatment for prevention of relapse was in Japan (23.4%) and the lowest in the US (9.0%).

Only 1% of patients specifically requested to be prescribed LAIs; 85% of these requests were granted by the psychiatrist.

#### Patient awareness and acceptance of LAIs

Overall, most (89.4%) LAI patients reported that they were “somewhat” (48.8%) or “completely” (40.6%) accepting of LAIs when they were first offered to them, whereas half (52.0%) of non-LAI patients reported that they would be “not at all” accepting of LAIs if their psychiatrist offered those treatment options to them (data not shown). Furthermore, most LAI patients had discussed LAIs with their psychiatrist (93.2%) and were aware that these treatments would remove the requirement of daily oral medication (87.0%). By contrast, around one-third of non-LAI patients had discussed LAIs with their psychiatrist (34.9%) and were aware of their benefits (38.0%). The same pattern was observed across individual countries.

#### Facilitators of and barriers to prescribing LAIs

Both LAI and non-LAI prescribers reported improved adherence to treatment and efficacy, less frequent dosing, and convenience for patients as facilitators of prescribing LAI treatment, although there were differences between prescribers in terms of aspects most frequently reported as facilitators (data not shown): compared with non-LAI prescribers, LAI prescribers reported improved health outcomes (19.5% vs. 46.3%) and adherence (62.3% vs. 90.2%) as facilitators significantly more frequently (*p*<0.0001, in both cases); compared with LAI prescribers, non-LAI prescribers reported improved efficacy as facilitator significantly more frequently (45.5% vs. 59.7%, *p*=0.025).

Both LAI and non-LAI prescribers reported patient fear/dislike of needles and the cost to the patient as the two most common barriers to prescribing LAIs, with 28% of all psychiatrists also reporting limited awareness of and knowledge about LAIs as a main barrier. However, the perception of which was the greater barrier differed: LAI prescribers reported patient fear/dislike of needles as the primary barrier to LAI therapy significantly more frequently than non-LAI prescribers (79.2% vs. 49.4%, *p*<0.0001); non-LAI prescribers reported cost to patient as the key barrier to LAIs significantly more frequently than LAI prescribers (54.6% vs. 35.7%, *p*=0.003).

In the US, LAI prescribers reported lack of awareness/knowledge of LAIs as a barrier to treatment significantly more frequently than non-LAI prescribers (45.9% vs. 6.7%, *p*=0.004). In China, compared with LAI prescribers, non-LAI prescribers reported complexity of prescription (11.1% vs. 27.3%, *p*=0.049) and inconvenience to patients (8.9% vs. 25.5%, *p*=0.038) as barriers to treatment significantly more frequently.

Some key country differences were also observed when considering all psychiatrists (i.e., regardless of LAI prescriber status): although improved adherence was the most frequently reported facilitator in all countries, this was lower in China (78.0%) and Japan (69.2%) vs. Spain (97.5%), France (95.2%), and the US (87.9%); dosing frequency was a facilitator reported by 84.0% of physicians in Spain, whereas in other countries this was considerably lower, with the lowest percentage in Japan (48.7%); improved health outcomes was a facilitator of LAI prescription in the US (53.2%) and Spain (49.4%), but not in the other countries.

In terms of barriers, patient fear/dislike of needles was the top barrier in all countries except China, where cost to patient was the most frequently reported barrier (72.0%); cost to patient was not considered an important barrier in other countries. Psychiatrist awareness, knowledge, and personal preference for oral compounds were reported as important barriers to LAI use in the US (74.2%), France (73.5%), and China (71.0%), but to a lesser extent in Spain (46.9%) and Japan (20.5%).

The top 3 facilitators of and barriers to prescribing LAIs, regardless of LAI prescriber status, are presented in Supplementary Table S3 (Additional file 3).

#### Severity of illness and level of impairment

Table 2 shows how psychiatrists rated their patients on the CGI severity of illness scale. Overall, significantly more LAI vs. non-LAI patients were classed by their psychiatrists as “moderately–amongst the most extremely ill” (*p*<0.0001).

**Table 2:** Psychiatrist-reported CGI severity of illness

	Overall		US		France		Spain		Japan		China	
	LAI	Non-LAI	LAI	Non-LAI	LAI	Non-LAI	LAI	Non-LAI	LAI	Non-LAI	LAI	Non-LAI
Number of patients	**1108**	**3042**	**241**	**842**	**333**	**450**	**410**	**392**	**83**	**416**	**52**	**943**
CGI severity of illness	***		***									
Normal, not ill at all, n (%)	25 (2.3)	115 (3.8)	4 (1.7)	28 (3.3)	3 (0.9)	6 (1.3)	10 (2.5)	10 (2.6)	7 (8.4)	29 (7.0)	1 (1.9)	42 (4.5)
Borderline mentally ill, n (%)	91 (8.2)	366 (12.0)	10 (4.2)	119 (14.1)	27 (8.1)	34 (7.6)	45 (11.3)	36 (9.2)	8 (9.6)	55 (13.2)	1 (1.9)	122 (12.9)
Mildly ill, n (%)	241 (21.8)	1000 (32.9)	47 (19.5)	227 (27.0)	51 (15.3)	88 (19.6)	85 (21.3)	89 (22.8)	29 (34.9)	145 (34.9)	29 (55.8)	451 (47.8)
Moderately ill, n (%)	391 (35.3)	998 (32.8)	102 (42.3)	299 (35.5)	108 (32.4)	153 (34.0)	134 (33.6)	151 (38.6)	28 (33.7)	157 (37.7)	19 (36.5)	238 (25.2)
Markedly ill, n (%)	246 (22.2)	399 (13.1)	55 (22.8)	121 (14.4)	102 (30.6)	116 (25.8)	78 (19.6)	73 (18.7)	9 (10.8)	20 (4.8)	2 (3.9)	69 (7.3)
Severely ill, n (%)	97 (8.8)	144 (4.7)	21 (8.7)	40 (4.8)	40 (12.0)	48 (10.7)	34 (8.5)	28 (7.2)	2 (2.4)	7 (1.7)	0 (0.0)	21 (2.2)
Among the most extremely ill patients, n (%)	17 (1.5)	20 (0.7)	2 (0.8)	8 (1.0)	2 (0.6)	5 (1.1)	13 (3.3)	4 (1.0)	0 (0.0)	3 (0.7)	0.0	0.0

***p≤0.0001

Blank cells indicate no change in sample size from the overall number of patients presented at the outset of the table. Where sample size varies, ‘n’ number is stated

*CGI* Clinical Global Impression, *LAI* long-acting injectable, *US* United States

There was also a significant difference in the US, where psychiatrists reported LAI patients as “moderately–amongst the most extremely ill” significantly more frequently than non-LAI patients (74.7% LAI vs. 55.6% non-LAI, *p*<0.0001). The proportion of LAI patients reported as “moderately–amongst the most extremely ill” was the highest in France (75.7%) and the lowest in China (40.4%).

Compared with non-LAI patients, psychiatrists reported significantly more LAI patients globally as having “moderate–very severe impairment” in a range of areas, including overall level of function (all *p*<0.0001, Table 3).

**Table 3:** Psychiatrist-reported impairment in QoL and different areas of functioning

	Overall		US		France		Spain		Japan		China	
	LAI	Non-LAI	LAI	Non-LAI	LAI	Non-LAI	LAI	Non-LAI	LAI	Non-LAI	LAI	Non-LAI
**Number of patients**	**1132**	**3105**	**251**	**884**	**335**	**451**	**410**	**392**	**84**	**434**	**52**	**944**
**QoL**	***		**						*			
No impairment, n (%)	85 (7.5)	295 (9.5)	9 (3.6)	60 (6.8)	24 (7.2)	22 (4.9)	39 (9.5)	28 (7.1)	8 (9.5)	37 (8.5)	5 (9.6)	148 (15.7)
Mild, n (%)	336 (29.7)	1318 (42.5)	74 (29.5)	322 (36.4)	73 (21.8)	144 (31.9)	135 (32.9)	161 (41.1)	28 (33.3)	202 (46.5)	26 (50.0)	489 (51.8)
Moderate, n (%)	526 (46.5)	1150 (37.0)	121 (48.2)	364 (41.2)	178 (53.1)	207 (45.9)	172 (42.0)	164 (41.8)	36 (42.9)	166 (38.3)	19 (36.5)	249 (26.4)
Severe, n (%)	172 (15.2)	298 (9.6)	42 (16.7)	112 (12.7)	59 (17.6)	73 (16.2)	58 (14.2)	32 (8.2)	11 (13.1)	26 (6.0)	2 (3.9)	55 (5.8)
Very severe, n (%)	13 (1.2)	44 (1.4)	5 (2.0)	26 (2.9)	1 (0.3)	5 (1.1)	6 (1.5)	7 (1.8)	1 (1.2)	3 (0.7)	0 (0.0)	3 (0.3)
**Social function**	***	(*n*=3104)	**									(*n*=943)
No impairment, n (%)	76 (6.7)	222 (7.2)	13 (5.2)	55 (6.2)	22 (6.6)	15 (3.3)	34 (8.3)	28 (7.1)	3 (3.6)	32 (7.4)	4 (7.7)	92 (9.8)
Mild, n (%)	310 (27.4)	1120 (36.1)	55 (21.9)	254 (28.7)	65 (19.4)	115 (25.5)	126 (30.7)	132 (33.7)	37 (44.1)	171 (39.4)	27 (51.9)	448 (47.5)
Moderate, n (%)	460 (40.6)	1220 (39.3)	108 (43.0)	383 (43.3)	158 (47.2)	192 (42.6)	153 (37.3)	150 (38.3)	26 (31.0)	189 (43.6)	15 (28.9)	306 (32.5)
Severe, n (%)	254 (22.4)	484 (15.6)	69 (27.5)	170 (19.2)	83 (24.8)	119 (26.4)	81 (19.8)	69 (17.6)	15 (17.9)	37 (8.5)	6 (11.5)	89 (9.4)
Very severe, n (%)	32 (2.8)	58 (1.9)	6 (2.4)	22 (2.5)	7 (2.1)	10 (2.2)	16 (3.9)	13 (3.3)	3 (3.6)	5 (1.2)	0 (0.0)	8 (0.9)
**Cognitive function**	****		**				*					
No impairment, n (%)	154 (13.6)	438 (14.1)	26 (10.4)	125 (14.1)	30 (9.0)	35 (7.8)	72 (17.6)	81 (20.7)	17 (20.2)	77 (17.7)	9 (17.3)	120 (12.7)
Mild, n (%)	414 (36.6)	1489 (48)	93 (37.1)	407 (46.0)	105 (31.3)	159 (35.3)	154 (37.6)	159 (40.6)	33 (39.3)	197 (45.4)	29 (55.8)	567 (60.1)
Moderate, n (%)	421 (37.2)	959 (30.9)	102 (40.6)	291 (32.9)	143 (42.7)	189 (41.9)	134 (32.7)	124 (31.6)	29 (34.5)	143 (33.0)	13 (25.0)	212 (22.5)
Severe, n (%)	133 (11.8)	199 (6.4)	28 (11.2)	53 (6.0)	55 (16.4)	64 (14.2)	44 (10.7)	26 (6.6)	5 (6.0)	15 3.5)	1 (1.9)	41 (4.4)
Very severe, n (%)	10 (0.9)	20 (0.6)	2 (0.8)	8 (0.9)	2 (0.6)	4 (0.9)	6 (1.5)	2 (0.5)	0 (0.0)	2 (0.5)	0 (0.0)	4 (0.4)
**Ability to meet own basic needs**			**				*					
No impairment, n (%)	242 (21.4)	732 (23.6)	49 (19.5)	201 (22.7)	62 (18.5)	109 (24.2)	116 (28.3)	125 (31.9)	10 (11.9)	58 (13.4)	5 (9.6)	239 (25.3)
Mild, n (%)	422 (37.3)	1364 (43.9)	84 (33.5)	369 (41.7)	126 (37.6)	160 (35.5)	141 (34.4)	153 (39.0)	38 (45.2)	218 (50.2)	33 (63.5)	464 (49.2)
Moderate, n (%)	361 (31.9)	793 (25.5)	90 (35.9)	242 (27.4)	108 (32.2)	130 (28.8)	118 (28.8)	92 (23.5)	32 (38.1)	132 (30.4)	13 (25.0)	197 (20.9)
Severe, n (%)	97 (8.6)	192 (6.2)	25 (10.0)	59 (6.7)	35 (10.5)	49 (10.9)	33 (8.1)	19 (4.9)	4 (4.8)	23 (5.3)	0 (0.0)	42 (4.5)
Very severe, n (%)	10 (0.9)	24 (0.8)	3 (1.2)	13 (1.5)	4 (1.2)	3 (0.7)	2 (0.5)	3 (0.8)	0 (0.0)	3 (0.7)	1 (1.9)	2 (0.2)
**Ability to work**			**						**			
No impairment, n (%)	71 (6.3)	294 (9.5)	7 (2.8)	76 (8.6)	17 (5.1)	24 (5.3)	41 (10.0)	44 (11.2)	3 (3.6)	30 (6.9)	3 (5.8)	120 (12.7)
Mild, n (%)	256 (22.6)	1007 (32.4)	45 (17.9)	240 (27.2)	61 (18.2)	88 (19.5)	96 (23.4)	96 (24.5)	29 (34.5)	196 (45.2)	25 (48.1)	387 (41.0)
Moderate, n (%)	326 (28.8)	929 (29.9)	84 (33.5)	239 (27.0)	86 (25.7)	107 (23.7)	101 (24.6)	107 (27.3)	34 (40.5)	172 (39.6)	21 (40.4)	304 (32.2)
Severe, n (%)	330 (29.2)	625 (20.1)	81 (32.3)	217 (24.6)	126 (37.6)	158 (35.0)	106 (25.9)	98 (25.0)	14 (16.7)	27 (6.2)	3 (5.8)	125 (13.2)
Very severe, n (%)	149 (13.2)	250 (8.1)	34 (13.6)	112 (12.7)	45 (13.4)	74 (16.4)	66 (16.1)	47 (12.0)	4 (4.8)	9 (2.1)	0 (0.0)	8 (0.9)
**Overall general health**		(*n*=3104)	***									(*n*=943)
No impairment, n (%)	175 (15.5)	512 (16.5)	18 (7.2)	140 (15.8)	56 (16.7)	97 (21.5)	91 (22.20)	91 (23.2)	7 (8.3)	45 (10.4)	3 (5.8)	139 (14.74)
Mild, n (%)	456 (40.3)	1527 (49.2)	109 (43.4)	394 (44.6)	122 (36.4)	162 (35.9)	153 (37.3)	169 (43.1)	40 (47.6)	245 (56.5)	32 (61.5)	557 (59.1)
Moderate, n (%)	415 (36.7)	909 (29.3)	98 (39.0)	296 (33.5)	138 (41.2)	155 (34.4)	132 (32.2)	113 (28.8)	31 (36.9)	128 (29.5)	16 (30.8)	217 (23.0)
Severe, n (%)	75 (6.6)	144 (4.6)	19 (7.6)	44 (5.0)	18 (5.4)	35 (7.8)	31 (7.6)	19 (4.9)	6 (7.1)	16 (3.7)	1 (1.92)	30 (3.2)
Very severe, n (%)	11 (1.0)	12 (0.4)	7 (2.8)	10 (1.1)	1 (0.3)	2 (0.4)	3 (0.7)	0 (0.0)	0 (0.0)	0 (0.0)	0 (0.0)	0 (0.0)
**Overall level of function**			**									
No impairment, n (%)	71 (6.3)	260 (8.4)	9 (3.6)	42 (4.8)	24 (7.2)	19 (4.2)	31 (7.6)	32 (8.2)	6 (7.1)	33 (7.6)	1 (1.9)	134 (14.2)
Mild, n (%)	370 (32.7)	1342 (43.2)	71 (28.3)	346 (39.1)	85 (25.4)	138 (30.6)	145 (35.4)	157 (40.1)	38 (45.2)	198 (45.6)	31 (59.6)	503 (53.3)
Moderate, n (%)	469 (41.4)	1145 (36.9)	117 (46.6)	365 (41.3)	140 (41.8)	190 (42.1)	160 (39.0)	153 (39.0)	32 (38.1)	173 (39.9)	20 (38.5)	264 (28.0)
Severe, n (%)	203 (17.9)	306 (9.9)	53 (21.1)	107 (12.1)	84 (25.1)	94 (20.8)	60 (14.6)	39 (10.0)	6 (7.1)	26 (6.0)	0 0.0)	40 (4.2)
Very severe, n (%)	19 (1.7)	52 (1.7)	1 (0.4)	24 (2.7)	2 (0.6)	10 (2.2)	14 (3.4)	11 (2.8)	2 (2.4)	4 (0.9)	0 (0.0)	3 (0.3)

*p≤0.05; **p≤0.01; ***p≤0.0001

Blank cells indicate no change in sample size from the overall number of patients presented at the outset of the table. Where sample size varies, ‘n’ number is stated

LAI: long-acting injectable; QoL: quality of life; US: United States

In the US, LAI vs. non-LAI patients were rated as “moderate–very severe” significantly more frequently for quality of life (QoL), social and cognitive functions, ability to work, and ability to meet own basic needs, as well as overall general health and overall level of function, whereas there were no significant differences between treatment groups in psychiatrist-reported impairment in France or China. In Spain, key differences were in cognitive function and ability to meet own basic needs, whereas in Japan it was the ability to work and QoL that showed the greatest differences between treatment groups.

#### Hospitalisations due to schizophrenia relapse and history of suicide attempt

Overall, LAI patients had experienced significantly more hospitalisations since diagnosis prior to the first LAI prescription vs. non-LAI patients (mean, 2.2 vs. 1.6, *p*<0.0001, Table 4). The highest mean number of hospitalisations prior to the first LAI prescription was reported in France (2.6) and the lowest in China (1.1).

**Table 4:** Number of hospitalisations due to schizophrenia relapse and number of suicide attempts

	Overall		US		France		Spain		Japan		China	
	LAI	Non-LAI	LAI	Non-LAI	LAI	Non-LAI	LAI	Non-LAI	LAI	Non-LAI	LAI	Non-LAI
Number of patients	**467**	**1496**	**66**	**211**	**123**	**172**	**216**	**209**	**18**	**162**	**44**	**742**
Number of hospitalisations prior to first LAI, mean (SD)	2.2 (2.9)***	1.6 (2.9)	1.7 2.9)	1.5 (3.3)	2.6 (3.2)*	3.8 (5.4)	2.3 (2.8)	2.6 (3.1)	1.4 (2.4)	1.2 (1.8)	1.1 (1.5)	0.9 (1.2)
Number of patients	**996**	**2783**	**205**	**723**	**296**	**401**	**390**	**375**	**84**	**434**	**52**	**922**
Number of suicide attempts, mean (SD)	0.4 (1.0)**	0.3 (1.3)	0.5 (1.0)	0.4 (2.0)	0.6 (1.1)	0.5 (1.3)	0.4 (0.9)	0.3 (0.7)	0.4 (0.9)*	0.2 (0.6)	0.1 (0.4)	0.2 (0.6)

*p≤0.05; **p≤0.01; ***p≤0.0001

Blank cells indicate no change in sample size from the overall number of patients presented at the outset of the table. Where sample size varies, ‘n’ number is stated

*LAI *long-acting injectable, *SD *standard deviation, *US* United States

Furthermore, more suicide attempts were reported in LAI vs. non-LAI patients (mean, 0.4 attempts vs. 0.3 attempts overall, *p*=0.002, Table 4). A similar trend was observed in Japan (0.4 LAI vs. 0.2 non-LAI, *p*=0.05). LAI patients had the highest number of suicide attempts in France (0.6 attempts) and the lowest in China (0.1 attempts).

## Discussion

The first aim of our survey was to assess discordance between psychiatrists and their patients with schizophrenia regarding disease management. Discordance was observed on their respective perception of severity of illness, level of improvement, level of adherence and reasons for non-adherence to treatment. Psychiatrists over-reported most treatment goals more frequently than patients. Previous studies identified discordance between patients and psychiatrists in the valuation of treatment goals for schizophrenia and this divide needs to be narrowed to achieve patient-centred care [6]. Indeed, discordance could lead to suboptimal treatment and poor patient outcomes, and future research should investigate factors associated with such discordance.

Our second aim was to understand the drivers of prescribing LAIs to patients with schizophrenia in a real-world clinical setting. We found that LAIs were more likely to be prescribed to patients with severe schizophrenia based on both clinical and psychosocial characteristics. Psychiatrist-reported data showed that LAI patients had been diagnosed for a longer period prior to data collection and the majority had more severe schizophrenia, as well as a history of more relapses on average. Overall, patients who were receiving a LAI were reported by psychiatrists as being more severe, as rated on the CGI severity of illness scale, with a greater number of hospitalisations in the previous 12 months and since diagnosis. LAI patients were more likely to experience cognitive symptoms, but less likely to experience symptoms relating to mood and sleep. LAI patients were also more likely to have received non-drug treatments for schizophrenia. A previous survey of almost 900 healthcare professionals across Europe found that 40% of them would choose LAI antipsychotics for first-episode patients, whereas 90% of them would select LAIs for chronic patients with two to five psychotic episodes [26]. Similarly, Sajatovitc et al. [27] reported that beyond non-adherence to treatment, experts (mainly psychiatrists) positioned LAIs as an appropriate treatment for patients with more severe illness or limited social support. Improving adherence to treatment and preventing relapses were also key drivers of prescribing LAIs by psychiatrists in our survey. These findings are in line with previous reports that experts considered LAI antipsychotics an appropriate option for individuals with known non-adherence or questionable adherence and for those at a particularly high risk of relapse [27].

However, compared with patients, at global level psychiatrists in the current survey tended to underestimate patients’ severity of illness and overestimate their adherence, which meant that more patients may have potentially benefited from switching to LAIs earlier. Previous evidence suggests that LAI antipsychotics are effective for treating first-episode psychosis and for early initiation of schizophrenia treatment [28]; often patients are non-adherent to oral antipsychotics early in the course of schizophrenia [29]. There is some evidence suggesting that earlier prescribing of a LAI may be associated with better outcomes (reduced relapse rates, greater symptom reduction, and higher remission rates) than oral medications [30], hence future research should evaluate whether earlier use of LAIs is warranted, as this strategy may help to avoid or minimise complications associated with poor adherence, relapses, and disease progression by improving medication adherence and reducing the risk of relapses.

At country level, while all psychiatrists (regardless of LAI prescriber status) in all countries reported improved adherence and dosing frequency as key facilitators of prescribing a LAI, the proportion of psychiatrists reporting these in each country differed, especially between Asia and Europe (from 69.2% in Japan to 97.5% in Spain for improved adherence and from 48.7% in Japan to 84.0% in Spain for dosing frequency). Similarly, while all psychiatrists in all countries reported patient fear/dislike of needles as a main barrier to prescribing a LAI, this differed from 50.0% of psychiatrists in China to 90.1% of psychiatrists in Spain. In contrast with China (72.0%), the US (47.6%), and Japan (43.6%), cost to patient was less frequently reported by psychiatrists as a barrier in France (8.4%) and Spain (11.1%). As in the study by Llorca et al. [31], country-level differences observed in the current survey are likely to have been influenced by country-specific healthcare systems, cultural differences, treatment centre-specific differences, and psychiatrist training and knowledge, all of which may have influenced LAI prescription patterns.

In the current survey, China had the lowest proportion of patients on a LAI (5.2%), which was considerably lower than in other countries (51.1% of patients in Spain and 42.6% in France). Interestingly, China also had the lowest proportion of patients who were reported as being “moderately–amongst the most extremely ill” (40.4%) by their psychiatrists and one of the lowest levels of agreement between psychiatrists and their patients on severity of illness (κ=0.182). A similar pattern was observed in Japan, which also had a low proportion of patients on a LAI (16.2%), suggesting that LAIs may be underutilised in China and Japan due to psychiatrists underestimating the severity of illness in patients with schizophrenia.

Several limitations should be considered in the interpretation of the results of our survey. Participating patients may have not reflected the general schizophrenia population, as they were visiting their physician, and may have been those who visited their psychiatrist more frequently and/or were more severely affected than those who did not consult as regularly. Also, the DSP was not based on a true random sample of psychiatrists or patients. While minimal inclusion criteria governed the selection of the participating psychiatrists, participation was influenced by their willingness to complete the survey. Lastly, the point-in-time design of this survey prevented any conclusions about causal relationships; however, identification of important associations was possible. Additionally, rates of adherence were reported as high compared to other literature. This could be due to the nature of matched physician and patients pairs, and therefore since these patients voluntarily completed a PSC they may be naturally more adherent patients.

Despite such limitations, real-world surveys play an important role in identifying areas of concern that are not usually addressed in randomised controlled trials (RCTs). Compared with RCT populations, real-world surveys include more heterogenous samples, which are more reflective of real-world clinical practice.

## Conclusions

In a real-world setting, LAIs were more likely to be prescribed to patients with severe schizophrenia, and poor clinical and psychosocial outcomes. Improving medication adherence and preventing relapses were among the most common reasons for prescribing LAIs. However, our findings indicated that psychiatrists may have underestimated their patients’ severity of illness and overestimated the level of improvement compared with patients, bringing into question whether more patients with schizophrenia could benefit from LAI therapy than currently do. Future research should further investigate whether earlier use of LAIs in the course of the disease is warranted, as this strategy may help to avoid or minimise complications associated with poor adherence, relapses, and the progression of disease to more severe schizophrenia.

## Supplementary information

**Additional file 1.** docx, supplemental table (Table S1. Example physician survey questions and answer options)

**Additional file 2. **docx, supplemental table (Table S2. Kappa scores level of agreement)

**Additional file 3.** docx, supplemental table (Table S3. Top 3 facilitators of and barriers to LAI prescription reported by psychiatrists (regardless of LAI prescriber status))

## Data Availability

All data, i.e. methodology, materials, data and data analysis, that support the findings of this survey are the intellectual property of Adelphi Real World. All requests for access should be addressed directly to Jason Shepherd at jason.shepherd@adelphigroup.com

## Abbreviations

BMI: Body mass index
CGI: Clinical Global Impression
DSP: Disease Specific Programme™
ER: Emergency room
LAI: Long-acting injectable
PRF: Patient record form
PSC: Patient self-completion form
QoL: Quality of life
RCT: Randomised controlled trial
US: United States
