# Supplementary material for: Patient-psychiatrist discordance and drivers of prescribing long-acting injectable antipsychotics for schizophrenia management in the real-world: a point-in-time survey
Source: BMC Psychiatry. 2022 Mar 17;22:187. doi: 10.1186/s12888-022-03846-x (PMC8932174; doi:10.1186/s12888-022-03846-x)
Supplement: Supplementary file 1 — Additional file 1. docx, supplemental table (Table S1. Example physician survey questions and answer options) [file 12888_2022_3846_MOESM1_ESM.docx]

# Additional file 1

**Table S1. Example physician survey questions and answer options**

| **Example Question** | **Example Answer Option** |
| --- | --- |
| 1. For the majority of your patients with schizophrenia, do you prefer oral or long-acting injection treatments for:  - Reducing relapse; - Reducing repeated relapse/hospitalisations; - Reducing healthcare resource utilisation; - Reducing healthcare costs; - Enhancing adherence to treatment. | - Long-acting injection; - Oral; - No difference between long-acting injection and oral. |
| 1. From your experience, which do you feel provides better efficacy for your patients with schizophrenia? | - Long-acting injections; - Oral antipsychotics; - No difference between long-acting injections and oral antipsychotics. |
| 1. From your experience, what do you feel are the main barriers to using long-acting injections over oral antipsychotics? | Answer options included: Cost to patient; patient fear/dislike of needles; adverse events; prescribing is more complex; etc. |
| 1. From your experience, what do you feel are the main facilitators to using long-acting injections over oral antipsychotics? | Answer options included: Improved adherence; improved efficacy; improved health outcomes; etc. |
