# Supplementary material for: Patient-psychiatrist discordance and drivers of prescribing long-acting injectable antipsychotics for schizophrenia management in the real-world: a point-in-time survey
Source: BMC Psychiatry. 2022 Mar 17;22:187. doi: 10.1186/s12888-022-03846-x (PMC8932174; doi:10.1186/s12888-022-03846-x)
Supplement: Supplementary file 2 — Additional file 2. docx, supplemental table (Table S2. Kappa scores level of agreement) [file 12888_2022_3846_MOESM2_ESM.docx]

# Additional file 2

**Table S2. Kappa scores level of agreement**

| **Kappa** | **Agreement** |
| --- | --- |
| **<0.0** | Less than chance/poor |
| **0.0–0.20** | Slight agreement |
| **0.21–0.40** | Fair agreement |
| **0.41–0.60** | Moderate agreement |
| **0.61–0.80** | Substantial agreement |
| **0.81–1.0** | Almost perfect agreement |
