# Supplementary material for: Patient-psychiatrist discordance and drivers of prescribing long-acting injectable antipsychotics for schizophrenia management in the real-world: a point-in-time survey
Source: BMC Psychiatry. 2022 Mar 17;22:187. doi: 10.1186/s12888-022-03846-x (PMC8932174; doi:10.1186/s12888-022-03846-x)
Supplement: Supplementary file 3 — Additional file 3. docx, supplemental table (Table S3. Top 3 facilitators of and barriers to LAI prescription reported by psychiatrists (regardless of LAI prescriber status)) [file 12888_2022_3846_MOESM3_ESM.docx]

# Additional file 3

**Table S3. Top 3 facilitators of and barriers to LAI prescription reported by psychiatrists (regardless of LAI prescriber status)**

|  | **Top 3 facilitators to using LAI over oral antipsychotics, n (%)** | **Top 3 barriers to using LAI over oral antipsychotics, n (%)** |
| --- | --- | --- |
| **Overall (n=466)** | Improved adherence  399 (85.6%) | Patient fear/dislike of needles  346 (74.3%) |
|  | Dosing frequency  303 (65.0%) | Cost to patient  181 (38.8%) |
|  | Patient convenience  260 (55.8%) | Personal preference for oral compounds  146 (31.3%) |
| **US (n=124)** | Improved adherence  109 (87.9%) | Patient fear/dislike of needles  102 (82.3%) |
|  | Dosing frequency  84 (67.7%) | Cost to patient  59 (47.6%) |
|  | Improved health outcomes  66 (53.2%) | Limited awareness of and knowledge about LAIs  51 (41.1%) |
| **France** **(n=83)** | Improved adherence  79 (95.2%) | Patient fear/dislike of needles  68 (81.9%) |
|  | Patient convenience  52 (62.7%) | Personal preference for oral compounds  39 (47.0%) |
|  | Dosing frequency  44 (53.0%) | Inconvenience for patient  36 (43.4%) |
| **Spain (n=81)** | Improved adherence  79 (97.5%) | Patient fear/dislike of needles  73 (90.1%) |
|  | Dosing frequency  68 (84.0%) | Personal preference for oral compounds  32 (39.5%) |
|  | Patient convenience  63 (77.8%) | Adverse events  26 (32.1%) |
| **Japan (n=78)** | Improved adherence  54 (69.2%) | Patient fear/dislike of needles  53 (68.0%) |
|  | Dosing frequency  38 (48.7%) | Cost to patient  34 (43.6%) |
|  | Improved health outcomes  31 (39.7%) | Adverse events  25 (32.1%) |
| **China (n=100)** | Improved adherence  78 (78.0%) | Cost to patient  72 (72.0%) |
|  | Dosing frequency  69 (69.0%) | Patient fear/dislike of needles  50 (50.0%) |
|  | Patient convenience  66 (66.0%) | Limited awareness of and knowledge about LAIs  48 (48.0%) |

LAI: long-acting injectable; US: United States
